# Physiological sub-typing of cold and freezing injury in *Triticum turgidum* subspecies with bioinformatic and expression characterization of glutathione reductase

**DOI:** 10.7717/peerj.21678

**Published:** 2026-09-07

**Authors:** Hulya Sipahi, Imren Kutlu, Razan Darwiche

**Affiliations:** 1 Department of Agricultural Biotechnology, Faculty of Agriculture, Eskisehir Osmangazi University, Eskisehir, Turkey; 2 Department of Field Crops, Faculty of Agriculture, Eskisehir Osmangazi University, Eskisehir, Turkey

**Keywords:** *Triticum turgidum*, Cold stress, Glutathione reductase, Tolerance mechanism, Antioxidant defense, Gene expressions

## Abstract

**Background:**

This study examined how different subspecies of *Triticum turgidum* (*T. durum, T. polonicum, T. turanicum*) respond to cold and freezing, assessing their water status, stress responses, and antioxidant system, with particular focus on the structure and function of glutathione reductase (TtGR).

**Methods:**

*TtGR* genes were first identified from the *T. turgidum* genome using publicly available genomic resources such as Ensembl Plants. Promoter regions (~2 kb upstream) were analyzed to identify cis-regulatory elements using PlantCARE. Gene classification was performed based on predicted subcellular localization and conserved domain features. Plants were subjected to cold acclimation and freezing treatments, and physiological, biochemical, and enzymatic parameters were measured.

**Results:**

Bioinformatics analyses identified four *TtGR* genes in the *T. turgidum* genome. The genes in two groups: cytosolic (Class I) and chloroplastic (Class II). Gene structure analysis showed a conserved exon–intron organization, while motif analysis confirmed the presence of Nicotinamide Adenine Dinucleotide Phosphate (NADPH)-binding and redox-active domains across all TtGR proteins. Several regulatory sequences in the promoters are involved in cold (DRE), abscisic acid (ABRE), and stress (STRE) responses, indicating that *TtGR* genes are dynamically regulated in response to environmental changes. Physiological analyses showed that freezing treatment reduces leaf water content in all genotypes, leading to turgor loss, hydrogen peroxide (H_2_O_2_) accumulation, and increased malondealdehyte (MDA) levels. However, tolerance mechanisms addressing water stress and membrane damage differ among genotypes. At the biochemical level, activation of the antioxidant defense system occurs in all genotypes. *T. turanicum* displays strong defense by significantly increasing enzyme activities, ensuring that the ascorbate-glutathione cycle continues under stress. By contrast, *T. polonicum*, although showing increased overall enzyme activities, experiences a dramatic drop in glutathione reductase (GR) activity at freezing temperatures, which restricts reduced glutathione (GSH) regeneration and creates a functional bottleneck in the antioxidant cycle. *T. durum* fails to sustain enzyme activities over the stress period, leading to an intermediate-sensitive response. Thus, whereas *T. turanicum* effectively maintains antioxidant function during freezing, *T. polonicum* and *T. durum* exhibit less efficient stress responses, either through enzymatic bottlenecks or a lack of sustained defense.

**Conclusions:**

One of the most striking findings of this study is the observed dissociation between *TtGR* gene expression levels and enzyme activities. Low temperature limits the link between transcription and enzyme function. The primary determinant of low-temperature tolerance in *T. turgidum* subspecies is the sustainability of GR enzyme activity and GSH regeneration under freezing conditions.

## Introduction

Cold temperatures (between 0 °C and +5 °C) and freezing temperatures (below −1 °C) are major abiotic stress factors that limit plant growth and development. These conditions disrupt the structural integrity of cell membranes, alter water balance, and reduce photosynthetic activity (Thomashow, 2010; Hasanuzzaman et al., 2015; Zhang et al., 2023). A common outcome of these processes is the accumulation of reactive oxygen species (ROS) within cells, leading to oxidative stress. This ROS accumulation results in irreversible cellular damage, including lipid peroxidation, protein oxidation, and membrane damage (Apel & Hirt, 2004).

Plants have developed complex defense mechanisms consisting of enzymatic and non-enzymatic antioxidant systems to limit the harmful effects of ROS. Within this system, enzymes such as superoxide dismutase, catalase, and peroxidase directly detoxify ROS, while antioxidant molecules such as glutathione and ascorbate maintain cellular redox balance (Gill & Tuteja, 2010; Hasanuzzaman et al., 2019). Glutathione (GSH) is considered a fundamental component in maintaining cellular redox balance due to its ability to directly neutralize ROS and its role in the ascorbate–glutathione cycle (Foyer & Noctor, 2011; Zechmann, 2014). Under stress conditions, an increase in glutathione disulfide (GSSG), which is the oxidized form of GSH, and a decrease in the GSH/GSSG ratio indicate disruption of cellular redox balance.

Glutathione reductase (GR; EC 1.8.1.7) is a key enzyme that uses NADPH to convert GSSG to its reduced form (GSH) (Foyer & Noctor, 2011). Therefore, GR is critical for maintaining cellular redox balance and supporting the ascorbate–glutathione cycle. Consequently, maintaining GR activity under abiotic stress is considered a key factor in plant resistance to oxidative damage (Yousuf et al., 2011).

Wheat (*Triticum aestivum* and related species), a primary global food source, is sensitive to extreme low-temperature stress. Cold stress adversely affects photosynthesis, growth, and yield (Guo, Liu & Chong, 2018; Zhang et al., 2023; Wang et al., 2022). Recent studies show that low temperatures affect not only oxidative metabolism but also genes involved in glutathione metabolism, carbon metabolism, and hormone signaling (Zhang et al., 2023).

Although studies on cold stress in *T. aestivum* are widespread, information is limited on the physiological and molecular responses to low temperatures in subspecies of *Triticum turgidum* (*T. turgidum* subsp. *durum*, *T. turgidum* subsp. *polonicum*, and *T. turgidum* subsp. *turanicum*). Specifically, the link between glutathione-based redox regulation and physiological responses under low temperatures in these subspecies remains unclear, representing a significant scientific and agricultural gap. The increasing frequency of sudden cold and freezing events due to climate change (IPCC, 2022) underscores the need to better understand wheat low-temperature tolerance. Comparing how different subspecies respond to stress is crucial for selecting genotypes in breeding programs.

This study aims to determine how cold and freezing stress affect seedling physiology in *T. durum*, *T. polonicum*, and *T. turanicum*. Specifically, it aims to examine the relationship between glutathione reductase gene expression and physiological and biochemical responses under low-temperature conditions. In addition, bioinformatic analyses of *GR* genes, including gene structure, conserved motifs, and promoter cis-regulatory elements, were performed to evaluate their potential regulatory roles in stress responses. By combining physiological, biochemical, and gene expression analyses, this study provides a broader understanding of glutathione-based redox regulation in these subspecies and helps to explain differences in their responses to cold and freezing stress.

## Materials and Methods

### Plant materials and low-temperature treatment

In this study, three subspecies of *Triticum turgidum* were used: *T. durum* (cv. Kunduru 1149), *T. polonicum* (landrace Mika), and *T. turanicum* (cv. Kamut, QK-77). The Mika genotype was collected from the Balıkesir region (Türkiye) and is conserved in the United States Department of Agriculture (USDA) gene bank. Kamut (QK-77) is a commercially registered *T. turanicum* variety.

Seeds were surface-sterilized with 10% (v/v) ethanol followed by 1.2% (w/v) sodium hypochlorite and rinsed thoroughly with sterile distilled water. Seeds were sown in 1.5 L pots containing a soil mixture (soil:sand:peat:perlite, 2:1:1:1, v/v/v/v). Fertilizers were applied per kg of soil as follows: 200 mg N ((NH_4_)_2_SO_4_), 125 mg K and 100 mg P (KH_2_PO_4_), 2.5 mg Fe (Fe-EDTA), and 5 mg Zn (ZnSO_4_·7H_2_O). Plants were grown for 35 days under controlled conditions (20/15 °C day/night, 14 h light/10 h dark photoperiod, light intensity of 370 μmol m^−2^ s^−1^, and 65% relative humidity). Field capacity was determined according to Danish et al. (2020) using the gravimetric difference method. The amount of water corresponding to 80% field capacity was calculated and maintained throughout the experiment by regular irrigation. Five seeds were initially sown per pot, and seedlings were thinned to three uniform plants per pot after emergence. Each treatment consisted of three independent biological replicates (*n* = 3), with each replicate represented by a separate pot. For cold acclimation, 35-days-old seedlings were subjected to a gradual temperature decrease (2 °C h^−1^) from 20 °C to 4 °C, followed by exposure to 4/2 °C (day/night) conditions for 3 days under the same photoperiod and light intensity. Freezing treatment was applied after acclimation by further decreasing the temperature at a rate of 2 °C h^−1^ until −10 °C was reached. Plants were maintained at −10 °C for 24 h under controlled conditions (14 h light/10 h dark, 370 μmol m^−2^ s^−1^, and 65% relative humidity). This freezing regime was selected based on our previously established controlled-freezing protocol for spring cereals (Kutlu, Gulmezoglu & Dokuyucu, 2026) and adapted for spring tetraploid wheat. The treatment was designed to induce measurable freezing injury while maintaining seedling viability after cold acclimation, thereby providing a standardized and reproducible stress treatment for comparative physiological and molecular analyses. Leaf samples were collected immediately after the completion of each treatment.

### RNA isolation, cDNA synthesis, and gene expression analysis of *GR* genes

Leaves (100 mg) were ground in liquid nitrogen, and RNA was extracted using the EZ-10 Spin Column Plant RNA Mini-Preps Kit (Bio Basic Inc., Markham, ON, Canada). RNA purity was verified by A260/A280 (1.9–2.1) and A260/230 (>2.0) ratios. cDNA was synthesized from 2 μg of RNA using the OneScript Plus cDNA Synthesis Kit (Applied Biological Materials Inc., Richmond, BC, Canada). The 20 μL reaction mixture contained the following: 10 μL SYBR Green mix (A.B.T.™, Ankara, Turkey), 1 μL forward primer (10 pmol), 1 μL reverse primer (10 pmol), 6 μL water, and 2 μL cDNA. Real-time quantitative polymerase chain reaction (qPCR) was performed using an Applied Biosystems 7500 Real-Time PCR System (Applied Biosystems, Foster City, CA, USA). Amplification conditions: an initial denaturation at 95 °C for 10 min, followed by 40 cycles consisting of 15 s at 95 °C, 30 s at the primer annealing temperature, and 30 s at 72 °C. GAPDH (GenBank: EU022331.1) was used as the reference gene for normalization of target gene expression (Kiarash et al., 2018). The stability of the reference gene was assessed based on its consistent expression across different conditions. Expression levels were determined using the 2^−ΔΔCT^ method (Livak & Schmittgen, 2001). Amplification specificity was confirmed by melt curve analysis. GR primers were designed using an online tool (Table 1). Primer specificity was verified by BLAST analysis against the *T. turgidum* genome to confirm that each primer pair uniquely matched its intended homoeolog. All experiments were conducted with three biological replicates and two technical replicates per biological replicate; the data were analyzed as the average of the technical replicates. Following analysis of variance (ANOVA), a *post hoc* Tukey test was performed in IBM SPSS Statistics 26.0 (IBM Corp., Armonk, NY, USA) to assess significant mean differences between the control and treatment groups, with a *p*-value of ≤0.05.

**Table 1:** Primer list used in this study.

Genes	Primer sequences (5′ -> 3′)
*GAPDH*	Forward: GGTGCCAAGAAGGTCATCAT
	Reverse: TGGTCATCAAACCCTCAACA
*TRITD4Av1G007820.3*	Forward: GCTGCTGTTTTCTCCCAAC
	Reverse: GAGTGGCTCTAAGAGGTCTG
*TRITD4Bv1G166520.2*	Forward: ACTGGAGGAGGTTGGGGTTA
	Reverse: TTGGTAGGTTCATCGCCGAA
*TRITD6Av1G218270.2*	Forward: CTATCTGTCGTCGGTCTCAG
	Reverse: TCTCAGCATCAACCACCAG
*TRITD6Bv1G217150.3*	Forward: CGCTATCTGTAGTCGGTCTC
	Reverse: TCTCAGCATCAACCACCAG

### Bioinformatics analyses

To identify *GR* genes, glutathione reductase protein sequences from *Arabidopsis thaliana* (https://www.arabidopsis.org/) and *Triticum aestivum* (Ensembl Plants) genome databases were used to perform a BLASTp search against the *Triticum turgidum* genome (genome assembly: Svevo.v1; https://plants.ensembl.org/) with an E-value cutoff of ≤1e−10s. To identify potential GRs, the conserved protein domains of all candidate genes were examined. After identifying potential *GR* genes, a phylogenetic tree was constructed including *T. turgidum*, *Sorghum bicolor*, *Arabidopsis thaliana*, *Zea mays*, *T. aestivum*, and *Oryza sativa* using the Neighbor-joining method with 1,000 bootstrap replications (Saitou & Nei, 1987). Multiple sequence alignments were performed using ClustalW to support this analysis.

Physicochemical properties, subcellular localization, conserved motifs, gene structures, promoter elements, and protein–protein interactions were analyzed using ExPASy, WoLF PSORT, MEME v5.4.1, GSDS v2.0 (Hu et al., 2015), PlantCARE (Lescot et al., 2002), and STRING, respectively. Heatmaps were generated with TBtools (Chen et al., 2020).

### *In silico* analysis of the expression profiles of *GR* genes

To investigate the tissue-specific and abiotic stress-dependent expression characteristics of *GR* genes, RNA-seq data from the *T. durum* ‘Svevo’ variety (PRJNA343594) and datasets from the study by Arenas-M, Castillo & Godoy (2022), which examined short-term heat stress during the early grain-filling stage, were utilized. All expression data were obtained from the Triticeae Expression Database; FPKM values were subjected to a log_2_(FPKM + 1) transformation to ensure comparability, and Z-score normalization was applied at the gene level. Using the obtained data, the expression profiles of *GR* genes were compared, and the results were visualized as heatmaps using TBtools.

### Physiological and biochemical analyses

#### Osmoregulation parameters

Three 1.5 cm-long discs were taken from leaf samples to determine relative water content (RWC) and loss of turgor (LOT); fresh weight (FW) was measured for each. The discs were then soaked in double-distilled water for 4 h to achieve full turgor, and turgor weights (TW) were recorded. The samples were then dried at 70 °C for 24 h to determine their dry weight (DW). Using these measurements, relative water content (RWC) and leaf water loss (LOT) were calculated as percentages (Gulen & Eris, 2003).

$\rm RWC = (FW-DW)/(TW-DW)\times 100$

$\rm LOT = (TW-FW)/TW\times 100$

The proline content was determined according to the method of Bates, Waldren & Teare (1973). Leaf samples (0.1 g) were homogenized with 3% sulfosalicylic acid and centrifuged at 6,000 rpm for 10 min. The resulting supernatant was reacted with ice-cold acetic acid and acid ninhydrin, incubated at 100 °C, and then cooled in an ice bath. After toluene extraction, the absorbance of the upper phase was measured at 520 nm, and the proline content was calculated using a standard curve. Results are expressed as µmol proline g^−1^ fresh weight.

The soluble protein (SPR) content in leaf samples was determined using the Bradford (1976) method with bovine serum albumin (BSA) as the standard. Absorbance values were measured at 595 nm, and protein content was calculated using the BSA standard curve.

#### Membrane injury parameters

Membrane stability index (MSI) analysis was performed on the leaves according to the method of Sairam, Deshmukh & Shukla (1997). Leaf samples were washed with double-distilled water to minimize the effect of cell sap, then cut into 1 cm pieces and transferred to tubes containing 25 mL of distilled water. After brief agitation, the initial conductivity (A) was measured. The same samples were heated to 100 °C for 30 min, then cooled, after which the total conductivity (B) was determined. The MSI value was calculated using these two measurements.

$\rm MSI = (1-A/B)\times 100$

To determine malondialdehyde (MDA) levels in leaves, the method described by Hodges et al. (1999) was used. First, approximately 0.1 g of the leaf sample was homogenized on ice with 1% TCA. The homogenate was then centrifuged at 12,000 rpm for 15 min. Next, the resulting supernatant was mixed with 20% TCA containing 0.5% TBA and incubated at 95 °C for 30 min. After incubation, samples were rapidly cooled and centrifuged again. Subsequently, the absorbance of the final supernatant was measured at 532 and 600 nm. MDA content was calculated as nmol g^−1^ fresh weight.

$\rm MDA\;content = [(A532-A600)\times extraction\;volume]\,/\,[155\times sample\;weight]$

#### Oxidative stress and antioxidant system

The hydrogen peroxide (H_2_O_2_) concentration was determined according to the method of Velikova, Yordanov & Edreva (2000). Leaf samples (100 mg) were homogenized with 0.1% TCA and centrifuged at 12,000×*g* for 15 min. The resulting supernatant was mixed with potassium phosphate buffer (pH 7.0) and KI, and absorbance was measured at 390 nm. The H_2_O_2_ content was calculated using a standard curve.

Antioxidant enzyme activities (SOD, APX, GR, and CAT) were determined according to the method of Cakmak & Marschner (1992). First, leaf samples (0.2 g) were homogenized with K–P buffer (pH 7.6). Next, the homogenate was centrifuged at 15,000× *g* for 20 min at 4 °C. The resulting supernatant was then used in the enzyme assays.

To determine superoxide dismutase (SOD) activity, 2.9 mL of K–P buffer (pH 7.6), 0.5 mL of sodium bicarbonate, 0.5 mL of methionine, 0.5 mL of nitroblue tetrazolium chloride (NBT), 0.5 mL of riboflavin, and 0.1 mL supernatant were added, gently mixed, and incubated in the dark for 10–15 min (until the color turned blue). Absorbance was measured at 560 nm. Enzyme activity was expressed as U mg^−1^ protein.

Ascorbate peroxidase (APX) activity was measured in a reaction mixture containing 0.7 mL of K–P buffer (pH 7.6), 0.1 mL H_2_O_2_, 0.1 mL ascorbic acid, and 0.1 mL enzyme extract. Enzyme activity was calculated based on the initial reaction rate and expressed as µmol mg^−1^ protein min^−1^.

GR activity was determined by monitoring the decrease in absorbance of NADPH at 340 nm for 1 min in a reaction mixture containing 0.7 mL K–P buffer (pH 7.6), 0.1 mL GSSG, 0.1 mL enzyme extract, and 0.1 mL NADPH. Enzyme activity was calculated based on the initial reaction rate after correction for non-enzymatic oxidation, using the extinction coefficient of NADPH (6.2 mM^−1^ cm^−1^), and expressed as nmol mg^−1^ protein min^−1^.

Catalase (CAT) activity was measured by tracking the decrease in absorbance at 240 nm for 1 min. The reaction mixture contained 0.8 mL K–P buffer (pH 7.6), 0.1 mL H_2_O_2_, and 0.1 mL enzyme extract. Activity was calculated as nmol mg^−1^ protein min^−1^ from the absorbance change at 25 °C over 1 min.

Reduced glutathione (GSH) content was determined using the method of Cakmak & Marschner (1992). Plant samples (0.1 g) were homogenized with 5% metaphosphoric acid and centrifuged at 12,000×*g* for 15 min. The supernatant was mixed with 150 mM phosphate buffer (pH 7.4) containing 5 mM EDTA, then 0.5 mL of 6 mM DTNB solution was added. After incubating at room temperature for 20 min, absorbance was measured at 412 nm. A standard curve was prepared using reduced glutathione.

Ascorbic acid (AsA) content was determined according to the method of Cakmak & Marschner (1992). This method is based on the reduction of Fe^3+^, added to the medium, to Fe^2+^ by ascorbic acid, followed by measurement of the red color from the Fe^2+^–bipyridine complex at 525 nm. To begin, leaf samples (0.5 g) were homogenized with 5% metaphosphoric acid and centrifuged at 4,000×*g* for 30 min. Then, 0.2 mL of the resulting supernatant was mixed with 0.5 mL of 150 mM phosphate buffer solution (pH 7.4) containing 5 mM EDTA and 0.1 mL of 10 mM DTT (1, 4-dithiothreitol). This mixture was incubated at room temperature for 15 min. After incubation, N-ethylmaleimide (NEM) was added to terminate the reaction. Next, to develop the color, 0.4 mL of 10% trichloroacetic acid (TCA), 0.4 mL of 44% orthophosphoric acid, 0.4 mL of 4% 2, 2′-bipyridine in 70% ethanol, and 0.2 mL of 3% ferric chloride (FeCl_3_) were sequentially added. The final mixture was incubated at 40 °C, and the absorbance was measured at 525 nm. The AsA content was determined using an L-ascorbic acid standard curve.

### Statistical analyses

The pot experiment was designed as a three-replicate factorial design comprising three temperature treatments (control, acclimatization, and freezing) and three wheat genotypes. The data were analyzed using analysis of variance (ANOVA). Differences between means were determined using the Tukey multiple-comparison test, with a significance level of *p* < 0.05.

Principal component analysis (PCA) was applied to evaluate relationships among physiological and biochemical parameters and to assess variation between treatments and genotypes. The data were standardized using Z-scores. Principal components were calculated based on the correlation matrix, and components with eigenvalues greater than 1 were considered. The results were evaluated using component loadings and biplots. Statistical analyses were performed using IBM SPSS Statistics v26.0 (IBM Corp., Armonk, NY, USA).

## Results

### Bioinformatics analyses of *GR* genes in *T. turgidum*

Four *GR* genes were identified in the *Triticum turgidum* genome (AABB) and designated as *TtGR1-A, TtGR1-B, TtGR2-A*, and *TtGR2-B* (Table 1), following the International Rules for the Genetic Nomenclature of Wheat (McIntosh et al., 1998). Phylogenetic analysis categorized these genes into two distinct clades corresponding to cytosolic Class I and chloroplastic Class II GRs (Fig. S1). Genes located on homoeologous group 4 chromosomes are designated as *TtGR1*, whereas those on group 6 chromosomes are designated as *TtGR2*; the letters “A” and “B” denote the subgenomes.

The TtGR proteins range from 497 to 550 amino acids in length, with molecular weights of 53.11 to 59.56 kDa (Table 2). TtGR2 isoforms exhibit acidic character, whereas TtGR1 isoforms are basic. All TtGR proteins show hydrophilic structures with negative GRAVY values.

**Table 2:** Genomic locations and physicochemical properties of glutathione reductases (GR).

Gene ID	Gene symbol	Chromosome: start-finish	Length (aa)	Molecular weight (kDa)	pI	GRAVY	Aliphatic index	Instability index (II)
TRITD4Av1G007820.3	*TtGR1-A*	4A: 16829077–16832920	550	59.36	8.36	–0.165	87.40	38.85
TRITD4Bv1G166520.2	*TtGR1-B*	4B: 566927412–566931144	550	59.56	7.67	–0.168	87.93	35.92
TRITD6Av1G218270.2	*TtGR2-A*	6A: 598291829–598297021	497	53.11	5.93	–0.040	92.01	21.07
TRITD6Bv1G217150.3	*TtGR2-B*	6B: 669008481–669013600	502	53.43	5.81	–0.049	90.72	21.69

The lower (more negative) GRAVY values of TtGR1s indicate that they exhibit high hydrophilicity in aqueous environments such as the chloroplast stroma. TtGR2s also have a soluble structure but exhibit slightly higher hydrophobicity compared to TtGR1s. TtGR2 isoforms showed higher aliphatic index values and lower instability index values compared to TtGR1 isoforms (Table 2).

**Figure 1:**
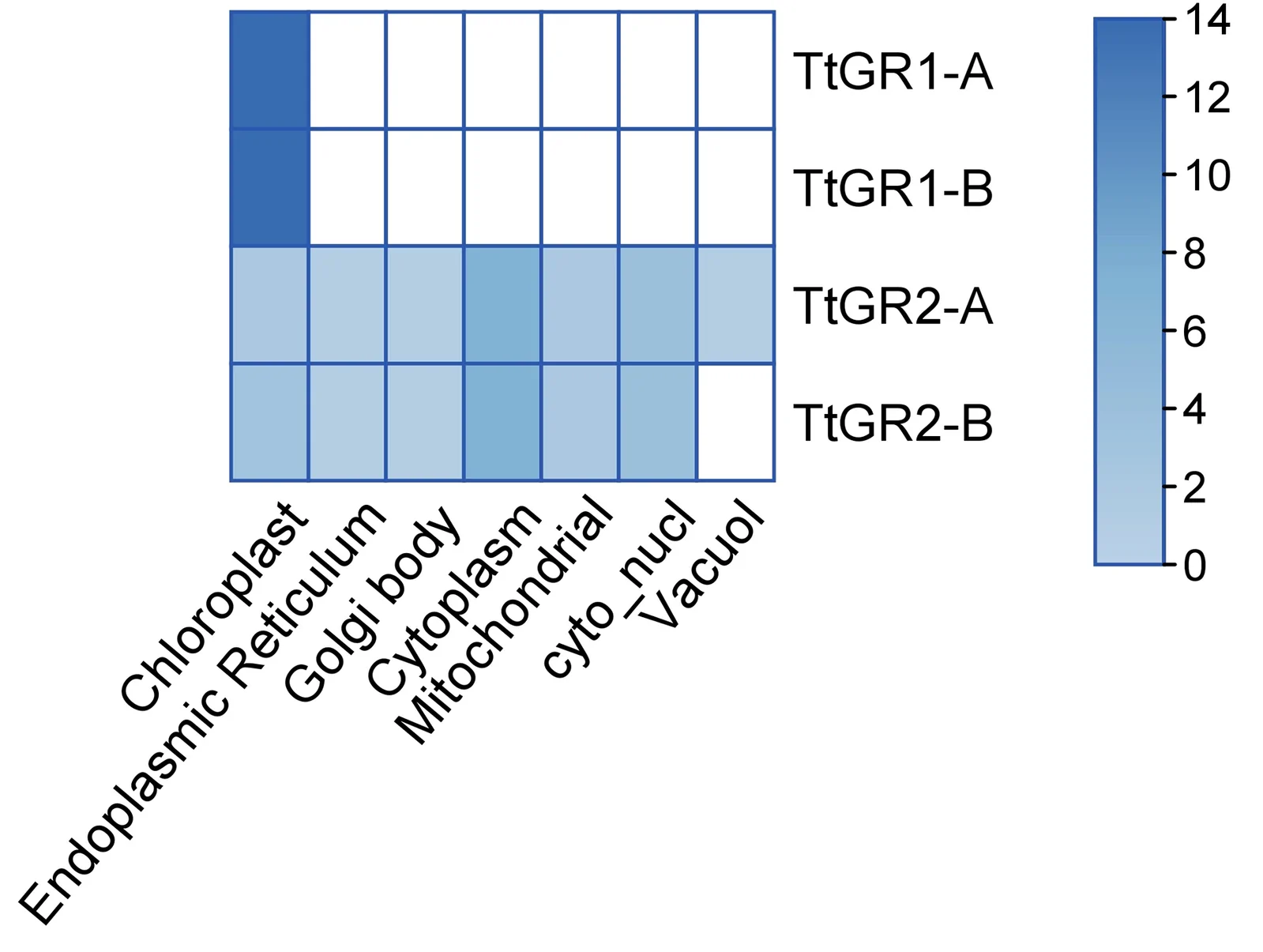
Intracellular localization of TtGRs.

Subcellular localization analyses indicated that TtGR1-A and TtGR1-B are chloroplast-localized Class II GRs, whereas TtGR2-A and TtGR2-B are cytosolic Class I GRs (Fig. 1). This classification is supported by the presence of an N-terminal transit peptide in TtGR1 isoforms.

Multiple sequence alignment showed that TtGR1 isoforms have an N-terminal alanine-, leucine-, and arginine-rich extension and are low in acidic amino acids, reflecting their conserved intracellular localization in Triticeae (Fig. S1). All TtGR isoforms contain the highly conserved FAD-binding motif (GXGXXG) in the N-terminal catalytic domain, as well as the redox-active Cys–X_4_–Cys motif required for GSSG reduction (Fig. S2). These conserved features indicate that core catalytic functions are preserved across all TtGR proteins.

**Figure 2:**
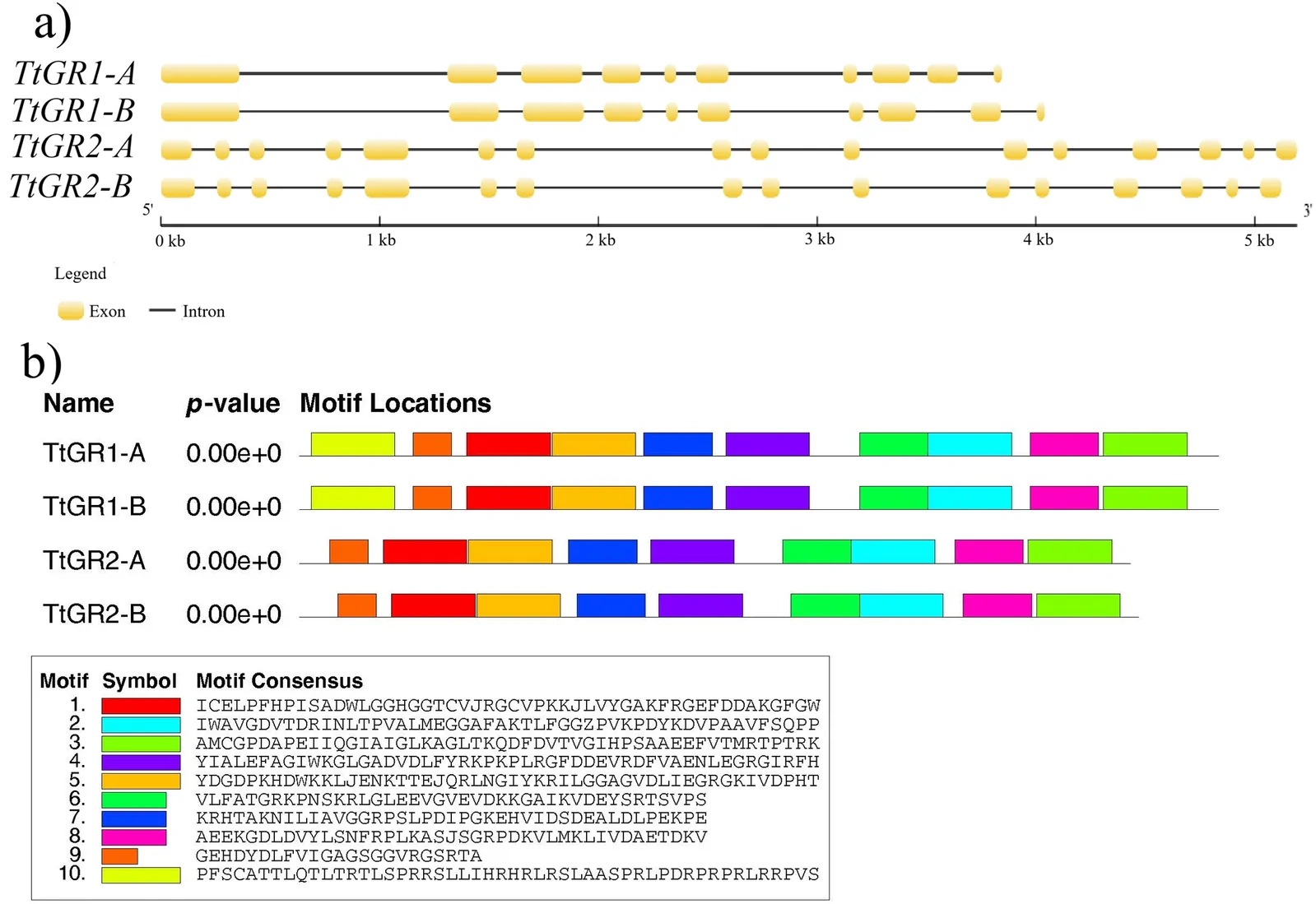
Genes and motifs structures of TtGR gene family.

Analysis of gene structures shows that *TtGR2-A* and *TtGR2-B* have more exons and introns (Fig. 2A). This matches the gene structure reported by Madhu et al. (2022) in bread wheat. Notably, the first intron in *TtGR1* genes is longer.

Motif analysis of TtGR proteins has revealed that both the TtGR1 and TtGR2 groups share key functional components specific to glutathione reductase enzymes, including oxidoreductase activity (Motif 3; PF00070), cofactor binding (Motifs 4, 5, 6, and 7; IPR036188), and dimerization (Motif 8; IPR016156) (Fig. 2B). The TtGR1 group is characterized by Motif 10, located in the N-terminal region of the sequence, which is absent in the TtGR2 group. In this context, it is thought that Motif 10 functions as an organelle targeting signal (transit peptide) that directs the protein to specific organelles. The fact that the *p*-value for all motifs is 0 confirms that both subgroups (TtGR1 and TtGR2) possess non-random, evolutionarily conserved GR protein architectures.

**Figure 3:**
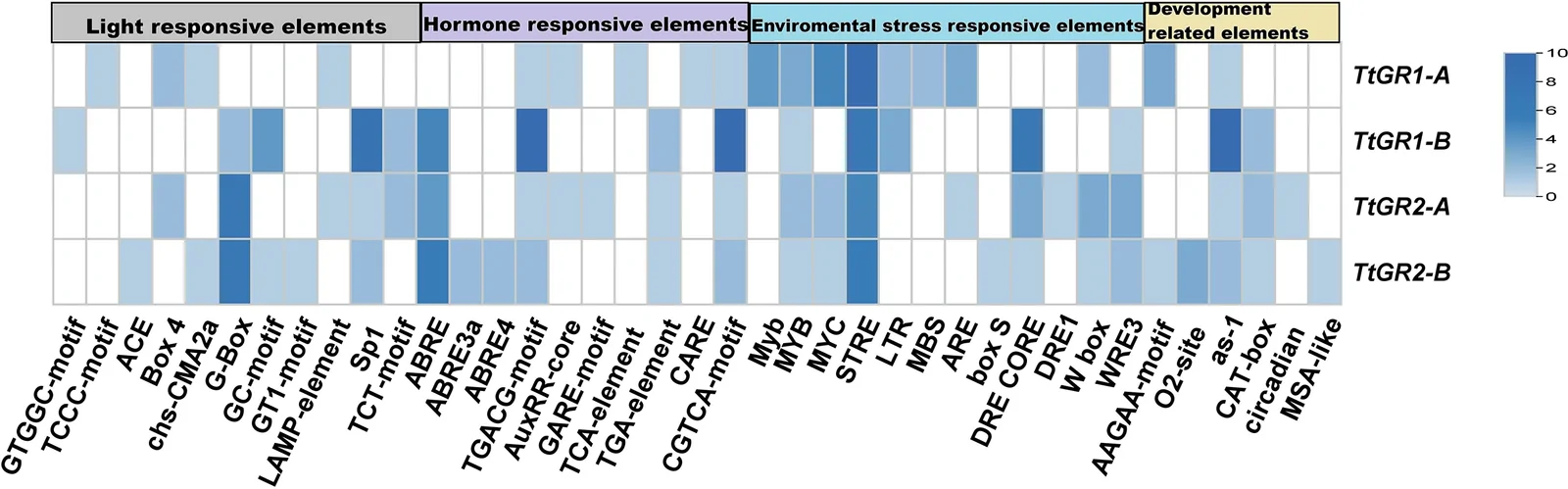
Cis-elements in *TtGR* gene promoters.

Cis-elements in the promoter regions of *TtGR* genes were examined to understand their transcriptional responses to light conditions, hormonal signals, and abiotic stress factors (Fig. 3). The presence of light-responsive cis-elements in *TtGR* promoters suggests that these genes have evolved to adapt to different light conditions and circadian rhythms. Specifically, the presence of numerous G-boxes and other light-responsive elements in *TtGR2* genes further supports this conclusion.

Turning to hormonal response motifs, the TGACG and CGTCA elements, previously characterized as methyl jasmonate (MeJA)-responsive cis-elements (Lescot et al., 2002), were identified in the TtGR1-B promoter, suggesting a potential involvement of this gene in MeJA-mediated signaling pathways. In contrast, the abundance of abscisic acid (ABRE), ABRE3a, and ABRE4 motifs in the *TtGR2-B* promoter indicates an involvement in abscisic acid (ABA)-mediated responses to drought and salinity stress. Collectively, this motif distribution indicates that *TtGR*s orchestrate specialized environmental stress responses, regulated by integrated hormone signaling pathways—including auxin, salicylic acid, and gibberellin—as well as antioxidant defense mechanisms.

*TtGR1-A* contains numerous stress response elements (STREs) and a Myb/MYB/MYC motif. The literature reports that these motifs play a role in defense against environmental stresses (Lescot et al., 2002). In addition, DRE CORE elements, previously associated with cold and drought stress (Lata & Prasad, 2011), were abundant in *TtGR1-B* and *TtGR2-A*. Similarly, the W-box and WRE3 motifs found in the *TtGR2-A/B* promoters are motifs known to provide transcriptional activation in pathogen and wounding responses (Rushton et al., 2010). Finally, the box S element in *TtGR2-B* is associated with pathogen responses.

Among the development-related motifs, the AS-1 (abscisic acid-responsive) motif is present in all genes but is particularly prevalent in *TtGR1-B*. It has previously been shown that this motif plays a role in oxidative stress signaling pathways and the salicylic acid response (Garretón et al., 2002). The O2-site (opaque-2) motif has been detected only in *TtGR2-B*. It has previously been shown that this motif regulates gene expression and protein synthesis during endosperm development in cereals (Lopes & Larkins, 1993). Therefore, it is thought that *TtGR2-B* may be associated with this developmental stage and seed stress tolerance. Additionally, the CAT-box and MSA-like motifs identified in this gene may be linked to regulatory processes associated with meristematic tissues and active cell division. Given that cell division is an energy-intensive process that generates reactive oxygen species (ROS), it is hypothesized that *TtGR2-B* may be regulated at this stage, in association with antioxidant defense mechanisms.

### *In silico* and real-time quantitative polymerase chain reaction gene expression analyses of *TtGR* genes

**Figure 4:**
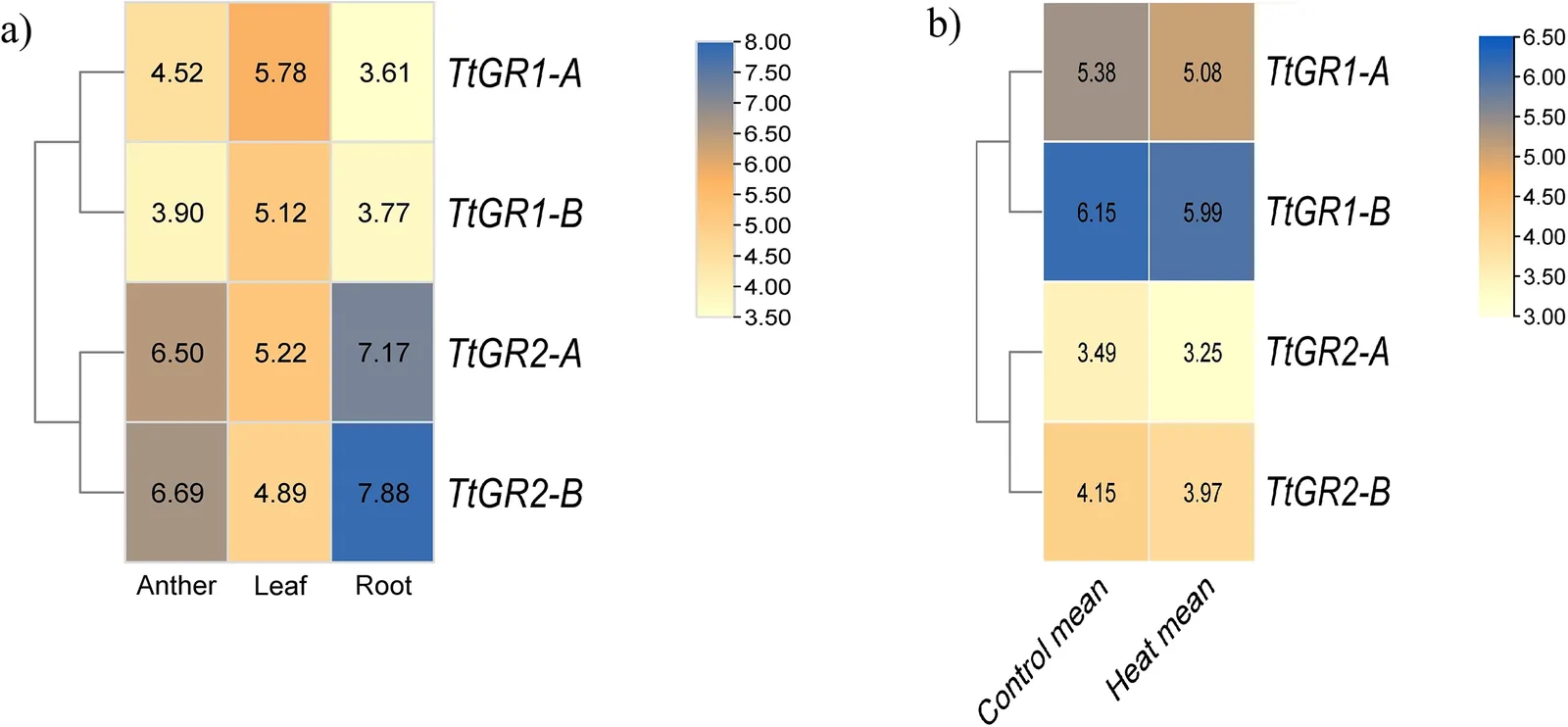
Tissue- and grain-development-specific expression profiles of TtGR genes. (A) Heat map showing the expression levels of *TtGR* genes in anther, root, and leaf tissues. (B) Heat map showing *TtGR* gene expression under control and short-term heat stress conditions during the early grain-filling stage.

*In silico* expression analysis of *TtGRs* in *T. turgidum* revealed distinct tissue- and heat-stress-associated expression patterns (Fig. 4). These results show that *TtGR* expression is tissue-specific (*TtGR1* in leaves, *TtGR2* in roots and anthers), and that short-term heat stress during early grain filling causes modest downregulation of all *TtGR* genes, with *TtGR1-B* being the highest and *TtGR2-A* the lowest under both control and stress conditions.

**Figure 5:**
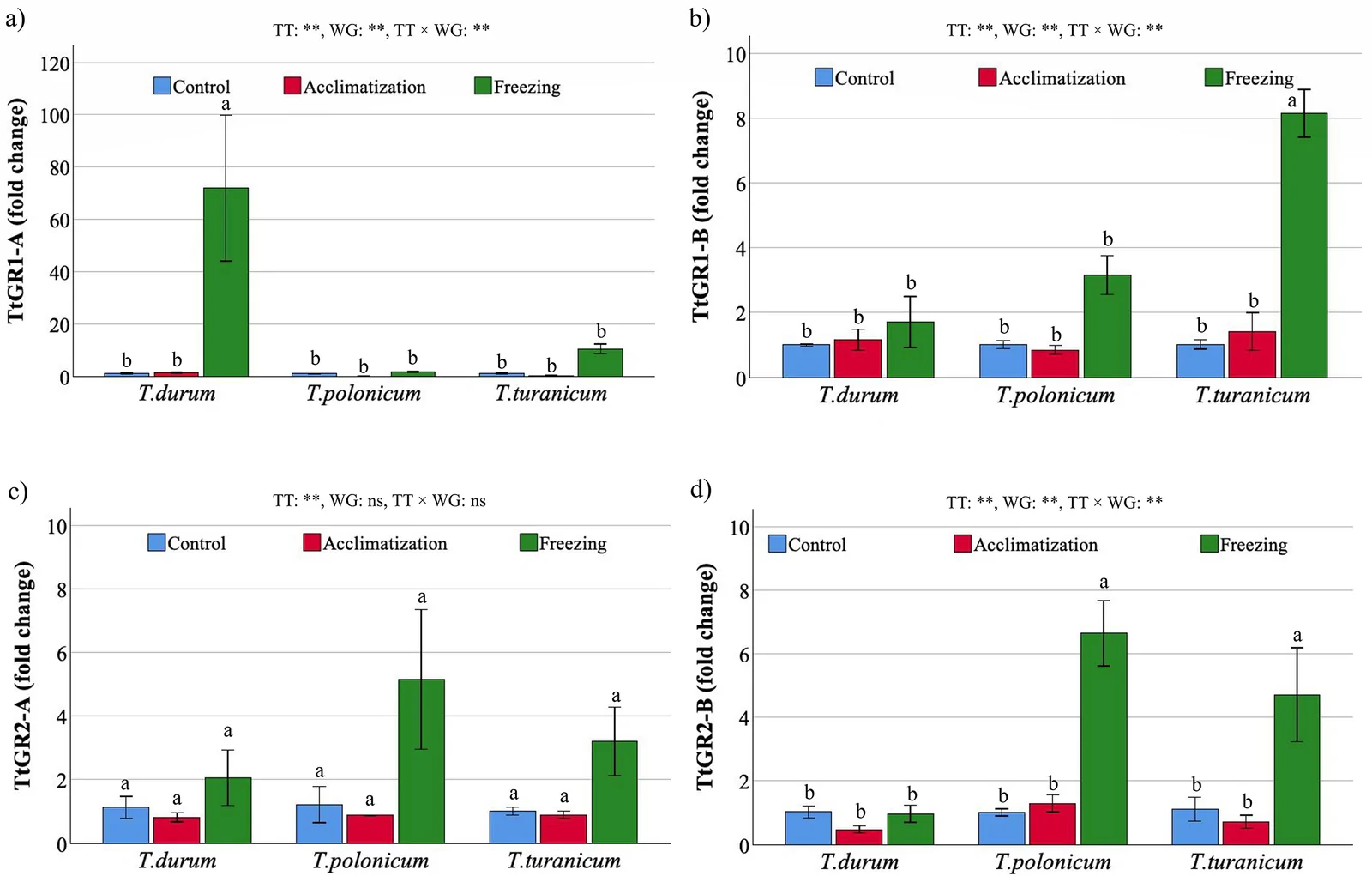
Expression levels of the *TtGR1-A* (A), *TtGR1-B* (B), *TtGR2-A* (C), and *TtGR2-B* (D) genes in *T. durum, T. polonicum*, and *T. turanicum* genotypes, as determined by RT-qPCR under control, acclimatization, and freezing conditions. Data are presented as mean ± SE (*n* = 3). Different letters indicate statistical significance (*p* < 0.05). TT indicates the main effect of cold stress, WG indicates genotype effect, and TT × WG indicates their interaction, based on ANOVA analysis (**p < 0.01; ns, not significant).

**Figure 6:**
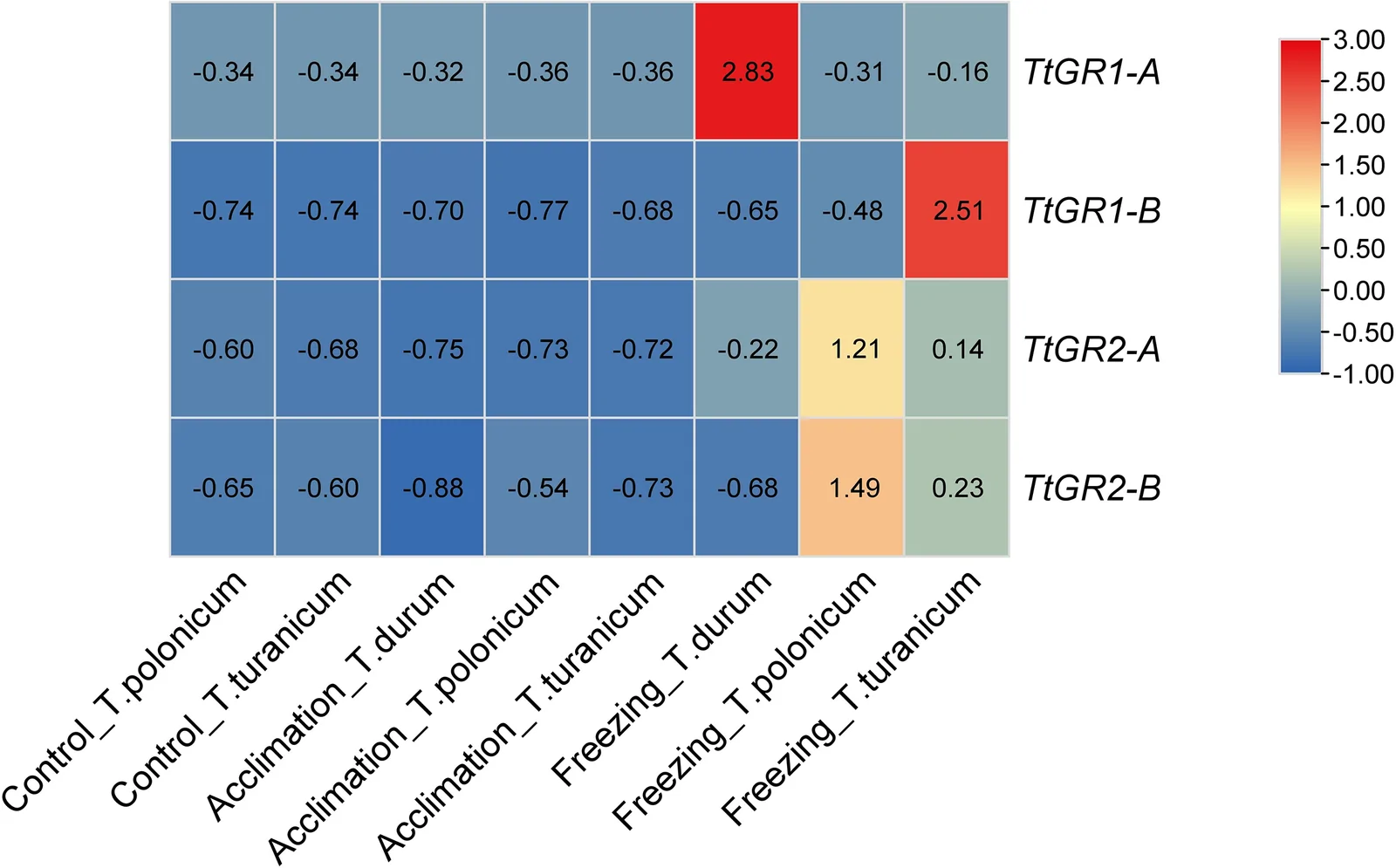
Heat map showing Z-score-normalized expression of *TtGR* genes based on qRT-PCR data for the *T. durum, T. polonicum*, and *T. turanicum* genotypes.

To elucidate the response of *TtGR* genes to cold stress, their expression levels under acclimation and freezing conditions were compared using real-time quantitative polymerase chain reaction (RT-qPCR) analysis (Figs. 5 and 6). Under acclimation conditions, *TtGR* gene expression stays at control levels or increases slightly, indicating the plant does not activate these genes during cold acclimation. However, under freezing conditions, *TtGR* expression was significantly higher than under normal conditions (green bars), suggesting that these genes may play a protective role against cold damage. Notably, the most pronounced change was observed in the *TtGR1-A* gene in *T. durum*, where expression levels increased 70–80-fold at freezing temperatures. Moreover, response patterns varied among genes and genotypes: while the largest increase was observed for *TtGR1-B* in *T. turanicum*, *TtGR2-A* and *TtGR2-B* showed a more moderate, balanced upregulation. Overall, *T. polonicum* and *T. turanicum* exhibited a stronger transcriptional response to cold than *T. durum*.

### Physiological and biochemical responses under low-temperature stress

In this study, the effects of cold and freezing stress on osmoregulation, membrane integrity, and the antioxidant system in *T. turgidum* subspecies were evaluated.

#### Osmoregulation parameters

**Figure 7:**
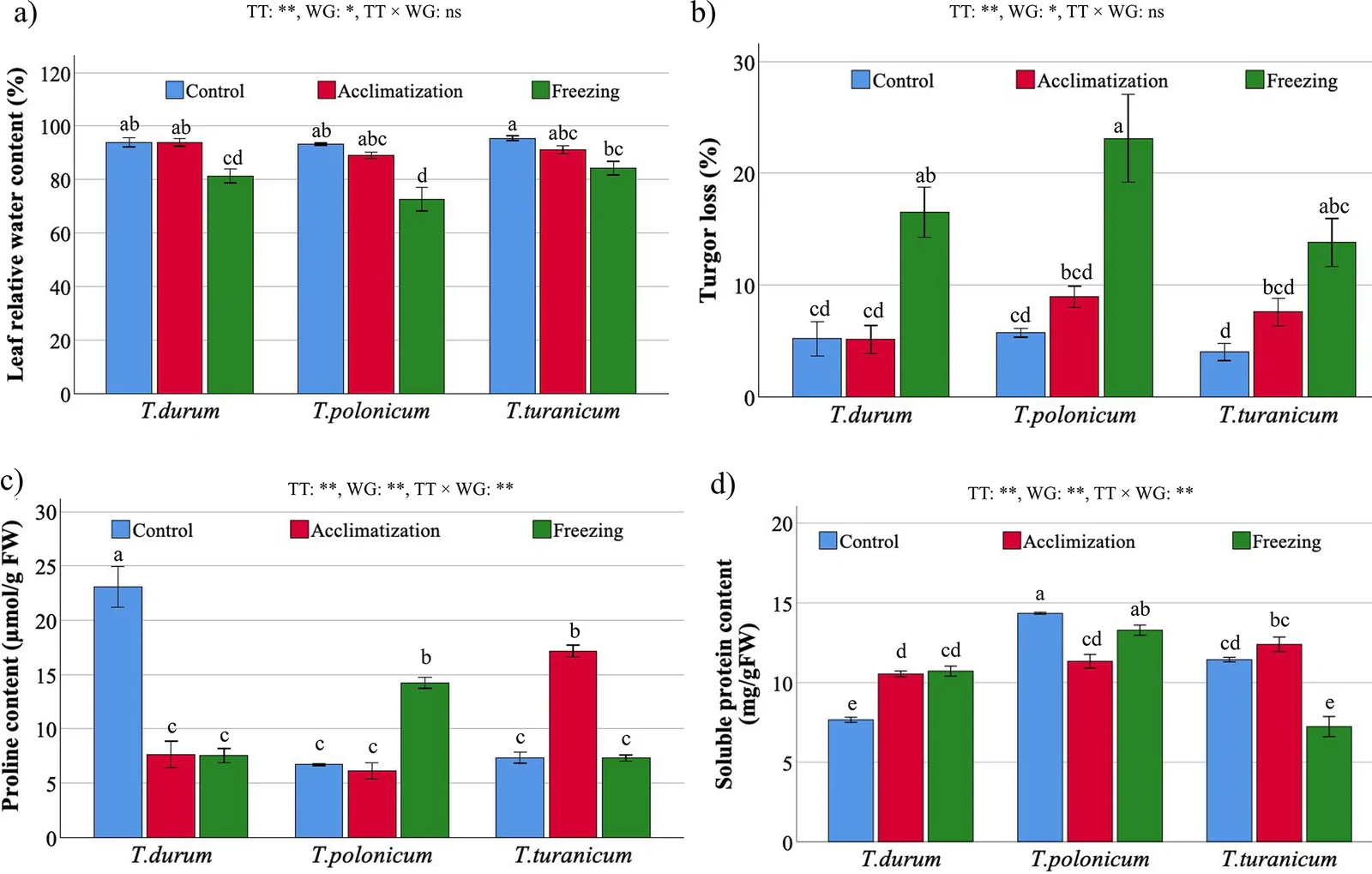
Osmoregulation parameters (A) leaf relative water content (RWC), (B) turgor loss (TL), (C) proline content, and (D) soluble protein content in *T. turgidum* subspecies under control, acclimation, and freezing conditions. Data are presented as mean ± SE (*n* = 3). Different letters indicate statistical significance (*p* < 0.05). TT indicates the main effect of cold stress, WG indicates genotype effect, and TT × WG indicates their interaction, based on ANOVA analysis (*p < 0.05; **p < 0.01; ns, not significant).

Two-way ANOVA indicated significant treatment and genotype effects on RWC and turgor loss, although treatment × genotype interaction was not significant (Figs. 7A, 7B). In contrast, proline and soluble protein contents were significantly affected by treatment, genotype, and their interaction (Figs. 7C, 7D).

The RWC of leaves decreased following freezing treatments (Fig. 7A). While the control and acclimation groups clustered in similar statistical categories, under freezing conditions, genotypes separated into distinct groups, indicating increased water loss. As stress increased, turgor loss also rose (Fig. 7B). Specifically, *T. polonicum* was statistically distinct from other genotypes, *T. turanicum* maintained low levels, and *T. durum* showed an intermediate response.

Proline response to acclimation and freezing treatments varied across genotypes (Fig. 7C). The highest proline content (23.07 µmol g^-1^ FW) was observed in *T. durum* under control conditions, but decreased under stress. In contrast, proline accumulation increased in *T. polonicum* under freezing and in *T. turanicum* during acclimation. Proline content varied depending on genotype and treatment (*p* < 0.05).

Changes in soluble protein content varied by genotype (Fig. 7D). In *T. durum*, protein content increased under stress conditions. In contrast, in *T. turanicum*, a significant decrease in soluble protein content was observed under freezing conditions. In *T. polonicum*, soluble protein content was intermediate compared with that of the other genotypes.

#### Membrane injury parameters

**Figure 8:**
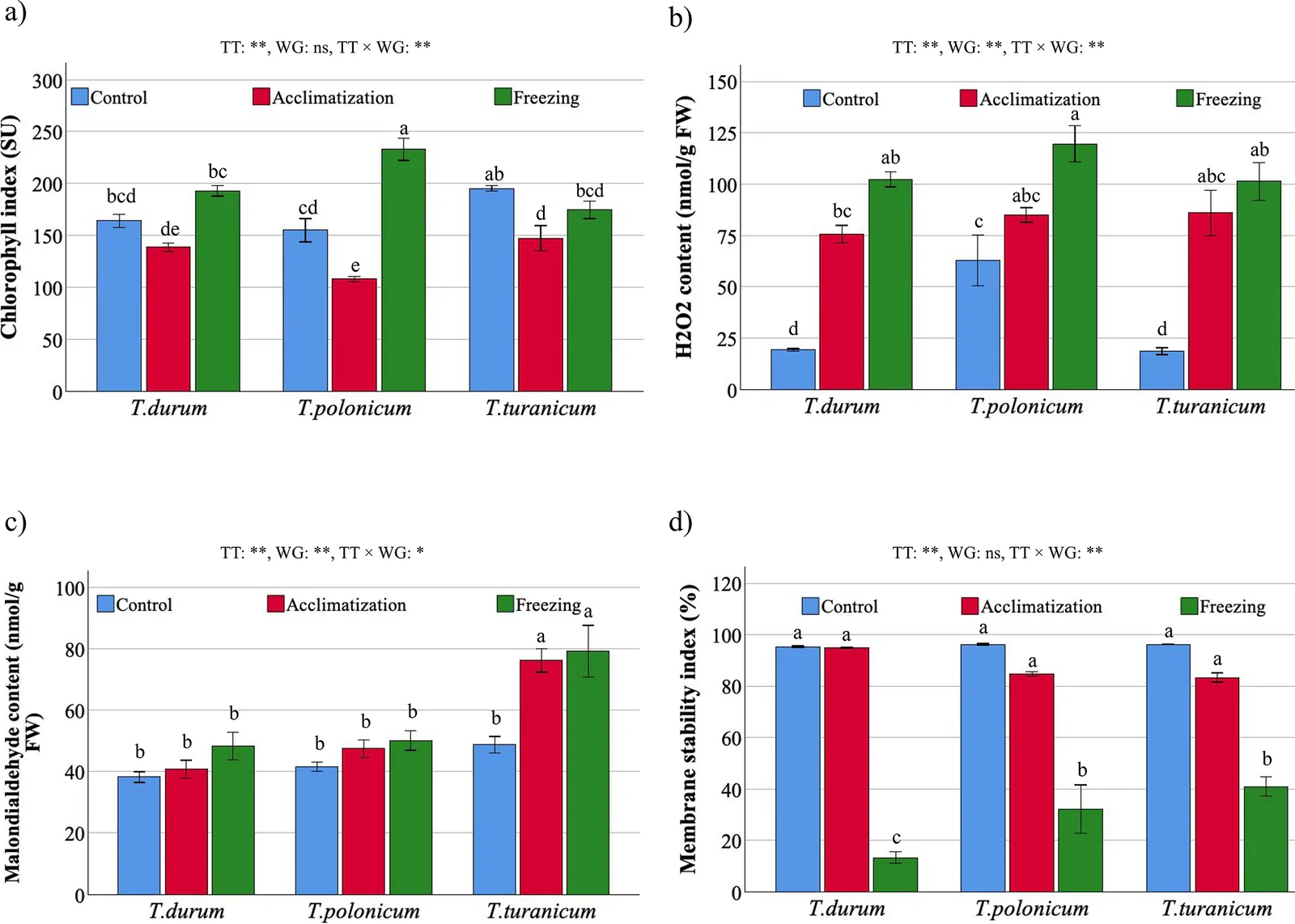
Membrane injury parameters (A) chlorophyll index, (B) hydrogen peroxide (H_2_O_2_) content, (C) malondialdehyde (MDA) content, and (D) membrane stability index (MSI) in *T. turgidum* subspecies under control, acclimatization, and freezing condit. Data are presented as mean ± standard error (SE) (*n* = 3). Statistical differences were evaluated at the *p* < 0.05 level. TT indicates the main effect of cold stress, WG indicates genotype effect, and TT × WG indicates their interaction, based on ANOVA analysis (**p < 0.01; *p < 0.05; ns, not significant).

Chlorophyll index was significantly influenced by treatment and the treatment × genotype interaction but not by genotype alone (Fig. 8A). Two-way ANOVA showed significant effects of both treatment and genotype on H_2_O_2_ content, whereas their interaction was not significant. MDA content was significantly affected by treatment, genotype, and their interaction, while MSI was significantly influenced by treatment and the treatment × genotype interaction but not by genotype alone (Figs. 8B–8D).

When the effects of cold and freezing stress treatments on the chlorophyll index were examined, it was determined that *Triticum* species exhibited different responses in terms of photosynthetic pigment stability (Fig. 8A). In particular, *T. polonicum* increased the chlorophyll index to the statistically highest level (*p* < 0.05) under freezing conditions. While a freezing-induced increase in chlorophyll was observed in *T. durum*, it was determined that the chlorophyll content in *T. turanicum* remained below the control level under both acclimation and freezing conditions.

H_2_O_2_ content increased gradually across all genotypes among control, acclimation, and freezing treatments (Fig. 8B). The highest values were observed under freezing conditions, with *T. polonicum* exhibiting the highest H_2_O_2_ content (119.6 nmol g^−1^FW). The acclimatization treatment increased H_2_O_2_ levels compared to the control group, but remained lower than those under freezing conditions. *T. durum* and *T. turanicum* showed a similar trend of increase.

Similarly, in terms of MDA content, *T. turanicum* showed higher values under both acclimatization (76.14 nmol g^−1^FW) and freezing (79.11 nmol g^−1^FW) conditions compared to *T. polonicum* and *T. durum*, which remained at lower levels (Fig. 8C).

Freezing stress disrupted cell membrane integrity, thereby increasing membrane permeability. MSI results indicated significant differences among genotypes (Fig. 8D). After freezing treatment, MSI values decreased in all genotypes, though the magnitude of the decrease varied. *T. turanicum* maintained the highest MSI (~40%) under freezing conditions, best preserving membrane stability, while *T. durum* had an MSI below 15%. By contrast, *T. polonicum* exhibited an intermediate MSI at approximately 30%. During acclimatization treatment, MSI values decreased only slightly, a change that was not statistically significant. This suggests that, during acclimatization, cellular adaptation mechanisms were activated rather than significant membrane damage occurring.

#### Antioxidant parameters

**Figure 9:**
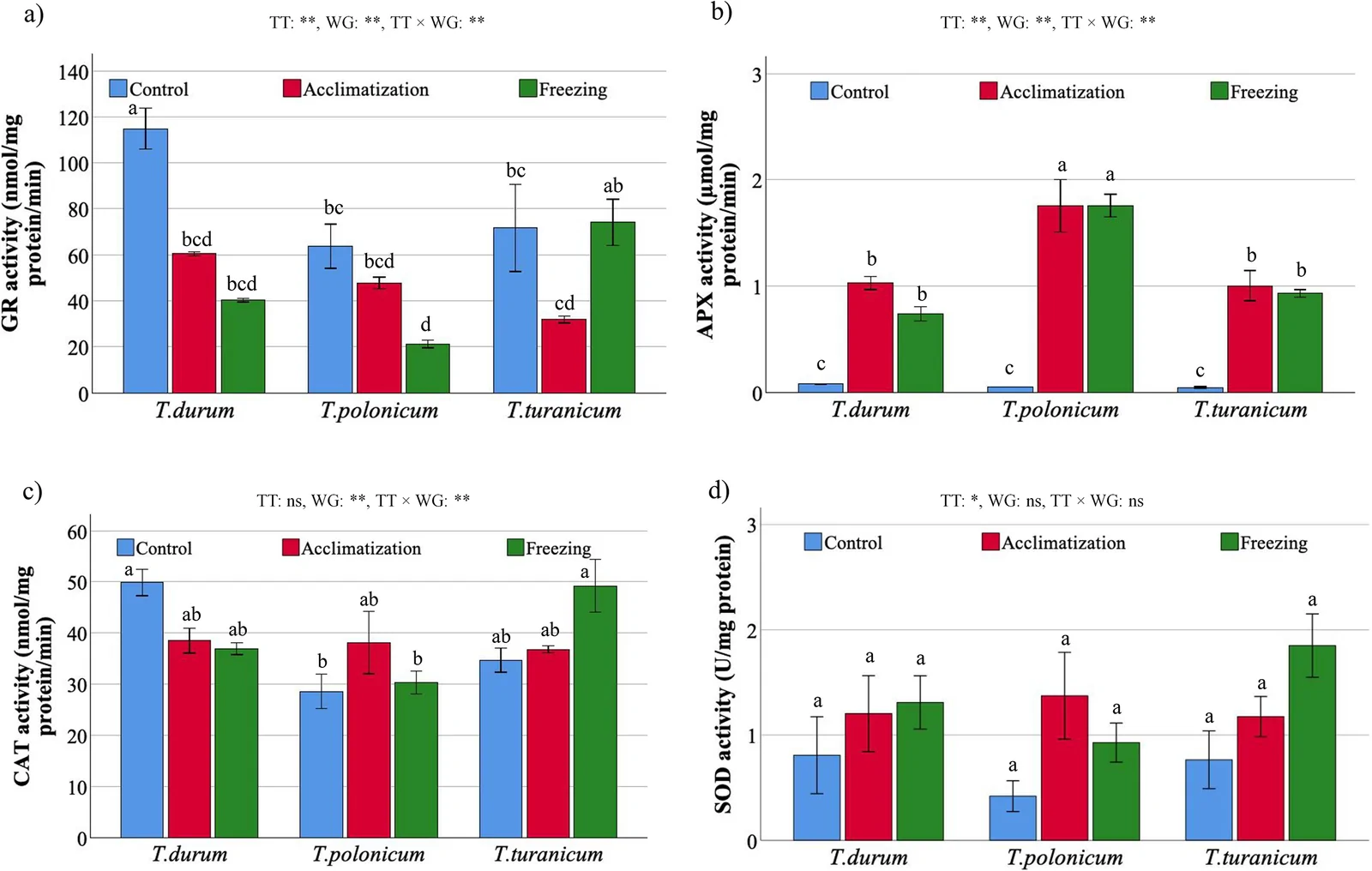
Changes in antioxidant enzyme activities in *T. turgidum* subspecies under control, acclimatization, and freezing: (A) glutathione reductase (GR), (B) ascorbate peroxidase (APX), (C) catalase (CAT), and (D) superoxide dismutase (SOD). Data are presented as mean ± standard error (SE) (*n* = 3). Statistical differences were evaluated at the *p* < 0.05 level. TT indicates the main effect of cold stress, WG indicates genotype effect, and TT × WG indicates their interaction, based on ANOVA analysis (**p < 0.01; *p < 0.05; ns, not significant).

**Figure 10:**
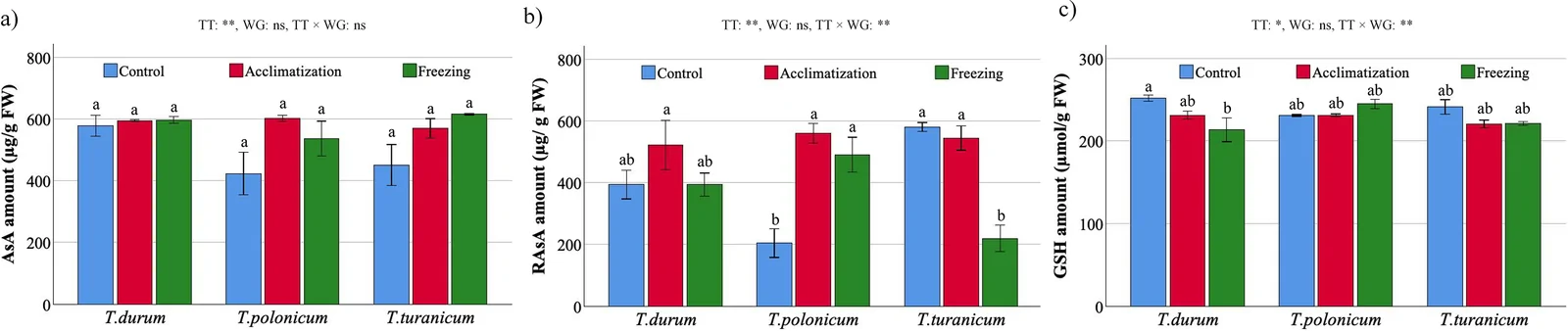
Non-enzymatic antioxidants (A) reduced ascorbate (AsA), (B) reduced ascorbate (RAsA), and (C) reduced glutathione (GSH) in *T. turgidum* subspecies under control, acclimatization, and freezing conditions. Data are presented as mean ± standard error (SE) (*n* = 3). Different letters indicate statistically significant differences among treatments within each genotype (*p* < 0.05). TT indicates the main effect of cold stress, WG indicates genotype effect, and TT × WG indicates their interaction, based on ANOVA analysis (*p < 0.05; ** p < 0.01; ns, not significant).

Two-way ANOVA revealed significant effects of treatment, genotype, and their interaction on GR, APX, and SOD activities. CAT activity was significantly affected by genotype and the treatment × genotype interaction, but not by treatment alone. AsA content was significantly influenced only by treatment, whereas RAsA and GSH contents were significantly affected by treatment and the treatment × genotype interaction but not by genotype alone (Figs. 9A–9D, Figs. 10A–10C).

Glutathione reductase (GR) activity, a key indicator of oxidative stress tolerance, showed significant differences among species under cold stress (Fig. 9A). During acclimatization, GR activity decreased across all three species compared to the control group. After freezing application, species-specific responses became even more pronounced. In *T. durum* and *T. polonicum*, GR activity continued to decrease, remaining below control levels. In contrast, in *T. turanicum*, GR activity increased again under freezing conditions, reaching approximately 15 nmol mg^−1^ protein min^−1^ and slightly exceeding control levels.

Acclimation and freezing treatments statistically significantly increased APX enzyme activity in all three wheat species (Fig. 9B). However, *T. polonicum* showed the highest APX activity under stress conditions, demonstrating a greater capacity for hydrogen peroxide detoxification than other species.

In interspecies comparison, *T. durum* showed relatively high basal CAT activity under control conditions (Fig. 9C). In contrast, *T. turanicum* showed an increasing trend in CAT activity under freezing conditions, reaching the highest values. *T. polonicum*, on the other hand, generally exhibited lower or moderate levels of CAT activity under all conditions and showed more limited variation compared to other species.

Superoxide dismutase (SOD) activity increased across all genotypes under acclimatization and freezing conditions (Fig. 9D). The highest value was observed in *T. turanicum*. However, the fact that all groups are in the same statistical letter group (a) indicates that this observed increase is not statistically significant (*p* > 0.05).

Ascorbic acid (AsA) content varied among genotypes under low-temperature conditions (Fig. 10A). Although the differences were not statistically significant, some trends were observed. In *T. turanicum*, AsA levels tended to increase under freezing conditions compared to the control. In *T. polonicum*, changes were more limited, while *T. durum* showed relatively stable or slightly reduced AsA levels under stress.

Levels of RAsA, the reduced ascorbate, differed significantly between species under cold and freezing conditions (Fig. 10B). In *T. polonicum*, the amount of RAsA increased compared to the control group under both acclimatization and freezing conditions. This suggests that the ascorbate pool in this species is less susceptible to oxidation. Meanwhile, in *T. durum*, the RAsA level increased during acclimatization but remained close to control levels under freezing conditions. In contrast, in *T. polonicum*, the RAsA content decreased significantly under freezing conditions and, reached its lowest values. Taken together, these findings indicate that *T. polonicum* can maintain its ascorbate pool in a more reduced state under freezing application and may be more effective in maintaining redox balance.

While GSH levels did not show statistically significant variation among genotypes, low temperatures and their interactions showed significant changes (Fig. 10C). In *T. durum*, GSH content decreased in parallel with decreasing temperatures, increased in *T. polonicum* under freezing stress, and in *T. turanicum*, although decreased compared to the control, it was preserved during acclimatization and freezing.

### Integrated analysis of molecular, physiological, and biochemical parameters

**Figure 11:**
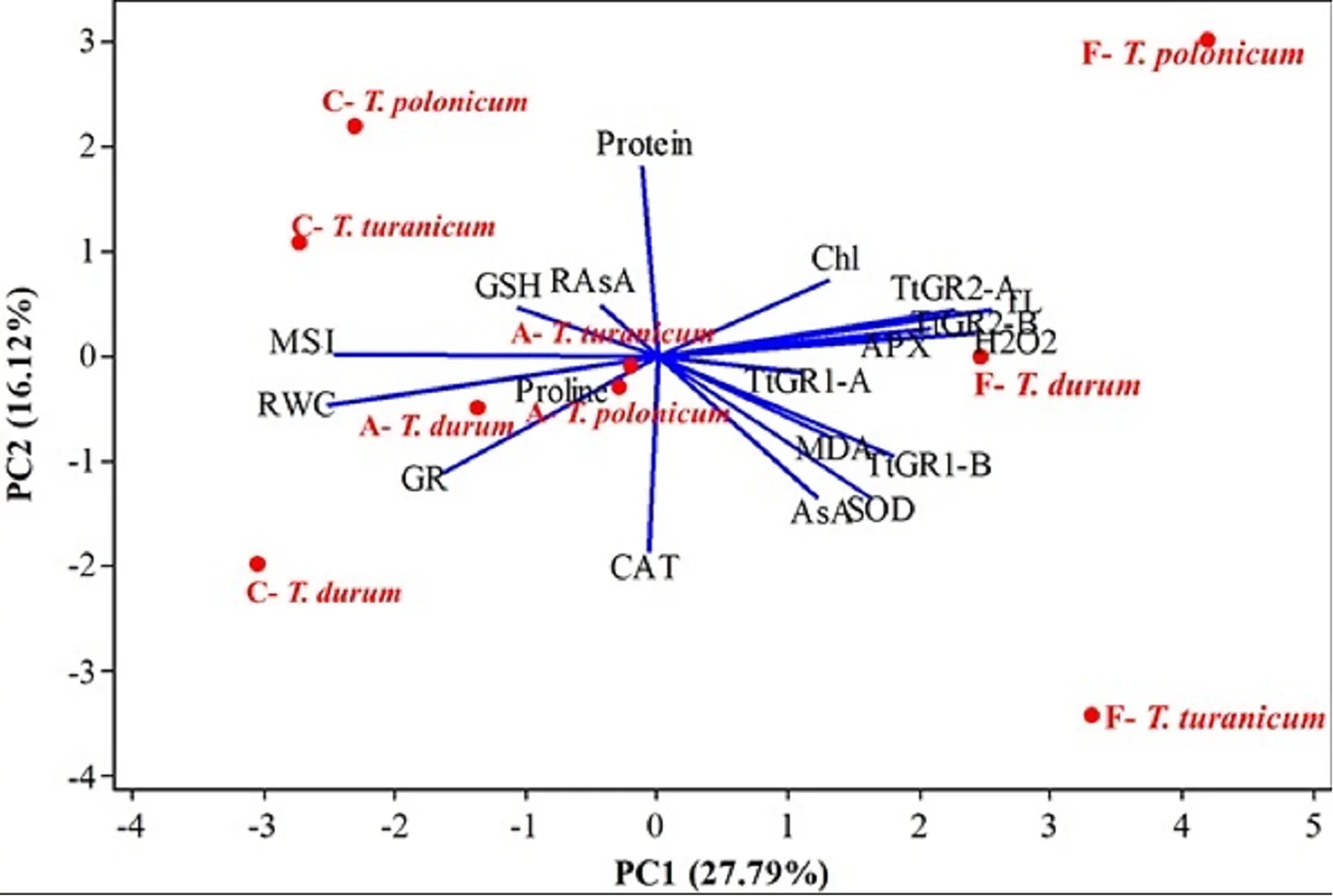
Principal component analysis (PCA) biplot of physiological and biochemical parameters measured under control (C), cold acclimatization (A), and freezing stress (F) conditions in *T. durum, T. polonicum*, and *T. turanicum*. The PC1 (27.10%) and PC2 (19.69%) axes explain 46.79% of the total variance. Vectors represent correlations between variables, and sample points represent genotype and treatments.

The PC1 axis separated samples by stress intensity (Fig. 11). Control and acclimatization groups were linked to RWC, GR, and GSH in the negative region. All genotypes under freezing stress shifted to the positive region, associated with H₂O₂ and MDA. The clustering of all genotypes near the origin during acclimatization suggests a similar metabolic response occurred before freezing. The 46.79% variance explained by PCA is typical for complex systems, where many physiological and biochemical parameters are assessed together.

Under freezing stress, genotypes diverged significantly along the PC2 axis. *T. turanicum* was associated with a strong antioxidant response, co-locating with SOD, CAT, and AsA, while *T. durum* was associated with higher oxidative stress, co-locating with MDA and H_2_O_2_. *T. polonicum* occupied an intermediate position, exhibiting a transitional profile associated with both antioxidant and other physiological parameters.

The distribution of *TtGR* genes on the PCA reveals functional divergence. TtGR2 isoforms co-located with H_2_O_2_ and APX, exhibiting a stress-induced response, while *TtGR1-B* is associated with SOD and AsA, playing a role in maintaining redox balance.

**Figure 12:**
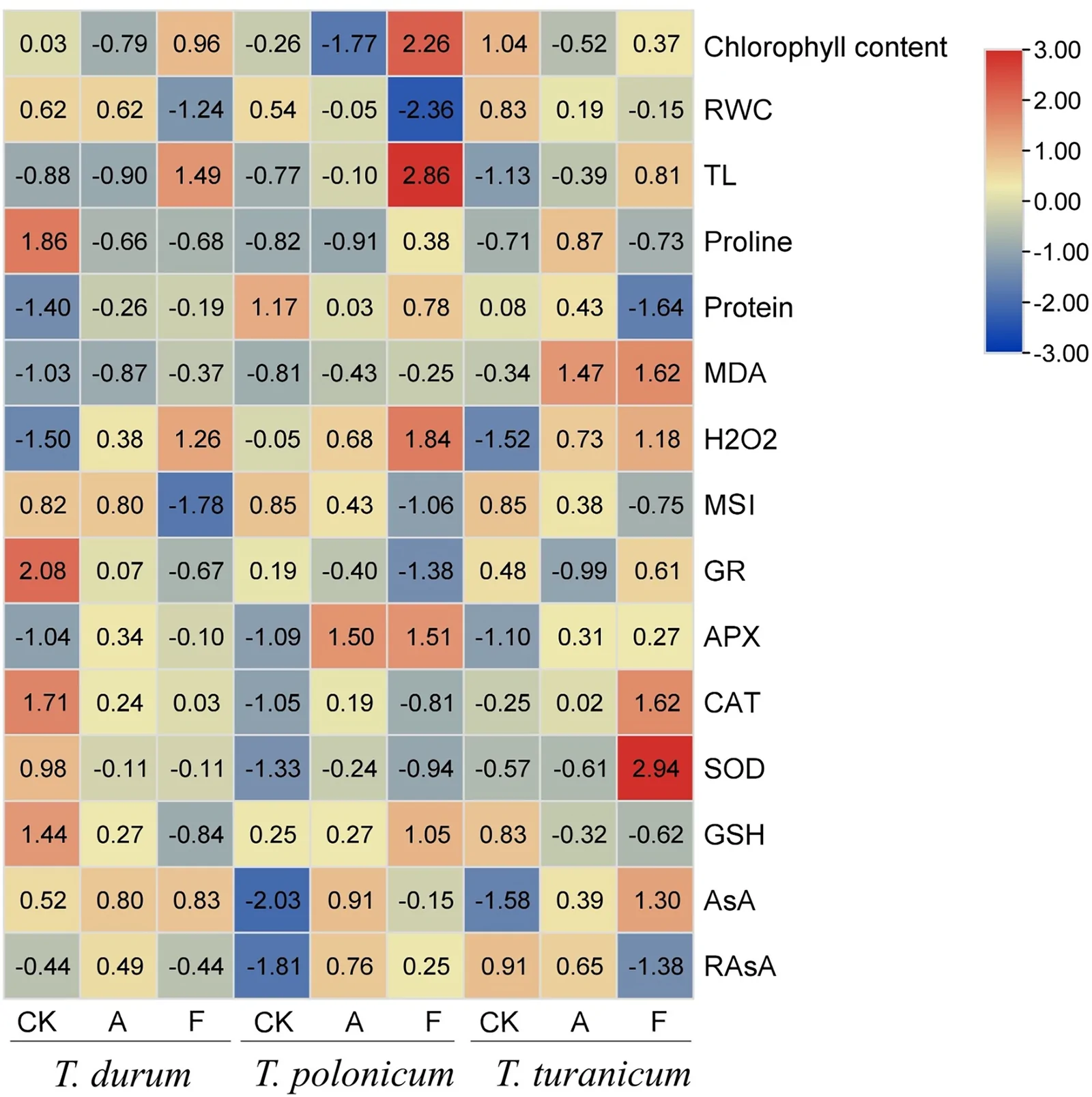
Heatmap representation of physiological and biochemical parameters measured under control (CK), acclimatization (A), and freezing (F) conditions in *T. turgidum* subspecies, based on Z-score normalization. Rows represent parameters, and columns represent genotype–treatment combinations. The color scale indicates the relative increase and decrease of each parameter.

The heat map findings (Fig. 12) generally align with the PCA results. The increase in H_2_O_2_ and MDA levels under freezing stress is associated with increased lipid peroxidation; however, the antioxidant response appears to vary by genotype. In *T. turanicum*, the increase in SOD and AsA is associated with strong ROS detoxification capacity, while the simultaneous increase in MDA suggests that membrane damage is not fully prevented. In *T. polonicum*, preservation of chlorophyll levels and increased proline accumulation suggest that this genotype may be advantageous for maintaining photosynthetic capacity and osmotic balance. In contrast, relatively low antioxidant activity, combined with high indicators of oxidative damage in *T. durum*, suggests that this genotype exhibits a more sensitive response to freezing stress.

## Discussion

This study evaluated the responses of *T. turgidum* subspecies to cold and freezing stress at the molecular, physiological, and biochemical levels. The bioinformatic characterization of *TtGR* genes, including their classification, structural features, and expression profiles, provided a genomic framework for interpreting stress responses. The results indicate that responses to low-temperature stress are genotype-dependent and closely associated with the regulation of cellular redox balance.

Bioinformatics analyses revealed that the four *TtGR* genes in the *T. turgidum* genome are divided into two main groups: cytosolic (Class I) and chloroplast (Class II). Physicochemical properties indicate that these isoforms exhibit distinct differences in cellular localization. In particular, the low instability index and high aliphatic index of the cytosolic TtGR2 isoforms suggest that these proteins may be structurally more stable under cytosolic conditions. In contrast, chloroplast TtGR1 isoforms are likely involved in maintaining stromal redox balance by detoxifying ROS generated during photosynthetic electron transport. Chloroplasts are among the organelles most affected by low-temperature stress and where ROS production is pronounced (Caverzan, Casassola & Brammer, 2016; Liang et al., 2026). The high hydrophilicity of TtGR1 isoforms may facilitate adaptation to stromal redox dynamics.

Cis-acting elements identified in *TtGR* promoters suggest that this gene family may be subject to stress-related transcriptional regulation rather than functioning solely as a passive antioxidant system. The presence of TGACG and CGTCA motifs in the *TtGR1-B* promoter is consistent with a potential role in jasmonate (MeJA)-mediated defense signaling, as these elements have been reported to be characteristic of jasmonate-responsive genes (Lescot et al., 2002). Jasmonate signaling, activated under cold stress conditions, interacts with the ICE–CBF–COR pathway and contributes to stress tolerance (Hu et al., 2013). In this context, *TtGR1-B* may participate at the interface of redox regulation and hormonal signaling. Overall, the promoter architecture of *TtGR* genes suggests a potential integration of redox balance with hormone-mediated stress signaling pathways and is consistent with the genotype-dependent transcriptional responses observed in this study.

The rapid accumulation of ROS under cold and freezing stress requires an effective antioxidant response (Kocsy, Galiba & Brunold, 2001; Suzuki & Mittler, 2006; Choudhury et al., 2017; Mittler, 2017). Consistent with this requirement, *TtGR* genes showed strong transcriptional induction under freezing conditions, indicating their involvement in redox regulation. However, gene expression did not directly correspond to enzyme activity.

Despite a 70–80-fold increase in *TtGR1-*A expression in *T. durum*, GR enzyme activity remained low under freezing conditions, suggesting that mechanisms acting downstream of transcription may influence enzyme activity. These mechanisms may include translational regulation, differences in protein abundance or stability, post-translational modifications, or cofactor availability under low-temperature stress (Piques et al., 2009; Ruelland et al., 2009; Vogel & Marcotte, 2012). In contrast, *T. turanicum* showed a coordinated increase in both *TtGR* expression and GR activity, indicating a more effective link between transcriptional and enzymatic responses. This suggests that maintaining GR activity may be more critical than transcriptional increase alone for cold tolerance. In *T. polonicum*, a decrease in GR activity despite relatively stable oxidative damage markers suggests that alternative protective mechanisms, such as non-enzymatic antioxidants or membrane stabilization, may contribute to stress tolerance. Overall, these findings indicate that genotype-specific differences in redox regulation depend not only on transcriptional responses but also on regulatory processes acting downstream of transcription.

Genotypic comparisons revealed that *T. polonicum* and *T. turanicum* exhibited stronger transcriptional induction of *TtGR* genes than *T. durum* under low-temperature stress. The higher *TtGR* expression observed in these genotypes highlights their potential for cold-tolerance breeding programs. However, this transcriptional advantage was not always reflected at the enzyme level, suggesting mechanisms acting downstream of transcription may contribute to the observed discrepancy between transcript abundance and enzyme activity under low-temperature conditions.

Cold and freezing stress affected the physiological and biochemical responses of *T. turgidum* subspecies, revealing clear genotype-dependent differences. The decrease in RWC at low temperatures is known to induce physiological drought under cold stress and disrupt water-uptake processes (Roychowdhury et al., 2025). This is further supported by PCA analysis, which shows a close relationship between RWC and MSI in our study and suggests a link between water status and membrane integrity. Relatively limited turgor loss and RWC decrease in *T. turanicum* indicate better maintenance of osmotic balance under stress. These findings align with previous studies observing that effective osmoregulation significantly contributes to membrane stability during freezing stress (Roychowdhury et al., 2025).

Proline is an important abiotic stress marker in plants that plays a role in osmotic adjustment, membrane stabilization and ROS scavenging (Caverzan, Casassola & Brammer, 2016; Roychowdhury et al., 2025). The increase in proline accumulation in *T. turanicum* during acclimation, as indicated by heatmap data, suggests that this species activates protective responses at an early stage of low-temperature expose. However, the subsequent decrease under freezing conditions indicates that this response is not sustainable. In contrast, *T. polonicum* reached higher proline levels under freezing conditions, suggesting a stronger response to severe cold rather than an early acclimation response. *T. durum* displayed a different pattern, with relatively high basal proline levels under control conditions that declined under stress, indicating that its proline-related defense capacity may be limited under freezing.

The increase in soluble protein content in *T. durum* under stress may reflect the synthesis of protective proteins and components of the antioxidant defense system. In contrast, *T. turanicum* showed a marked decrease in soluble protein content under freezing conditions Fig. 7D, and its position in the PCA biplot was oriented opposite to the protein vector, suggesting reduced cellular protein stability under severe cold. This decline may be associated with accelerated protein degradation (Gangopadhyay, 2015), suppressed translation, and oxidative protein damage caused by ROS (Roychowdhury et al., 2025), indicating substantial metabolic disruption under freezing stress.

Distinct adaptation strategies emerged among genotypes. *T. polonicum* exhibited a response profile characterized by relatively high chlorophyll levels and low accumulation of MDA and H_2_O_2_, suggesting that the integrity of the photosynthetic apparatus is maintained under stress. This implies a functional relationship between limiting oxidative damage and maintaining pigment stability. However, *T. polonicum* also showed relatively low GR activity, indicating that the glutathione-dependent component of the ascorbate–glutathione cycle may be more limited in this genotype compared to others.

From the perspective of the ascorbate–glutathione cycle, clear differences among genotypes were observed. *T. polonicum* appears to maintain redox balance by preserving the ascorbate pool in its reduced form. However, low GR activity in *T. polonicum* may limit GSH regeneration and reduce the glutathione cycle’s sustainability. This supports the view that stress tolerance depends not only on antioxidant levels but also on the ability to continuously replenish these components (Foyer & Noctor, 2011). Nevertheless, the relatively low MDA accumulation observed in *T. polonicum* suggests that this limitation does not directly translate into severe lipid peroxidation, indicating that alternative protective mechanisms, such as non-enzymatic antioxidants or membrane stabilization, may contribute to stress protection in this genotype.

Ascorbate (AsA) levels also support the genotype-specific differences in redox regulation observed in this study. Although the changes were not statistically significant, *T. turanicum* tended to maintain higher AsA levels under freezing conditions, suggesting a better preservation of the total ascorbate pool. This may contribute to the continued functioning of the ascorbate–glutathione cycle under stress. In contrast, the comparatively lower or less stable AsA levels in *T. polonicum* indicate that its total ascorbate pool may be more limited, despite showing lower oxidative damage markers. Taken together, these observations suggest that the efficiency of the antioxidant system under low-temperature stress depends not only on glutathione regeneration but also on the availability of reduced ascorbate.

Glutathione (GSH) is a key component of the non-enzymatic antioxidant defense system that maintains cellular redox homeostasis by detoxifying reactive oxygen species generated under low-temperature stress (Fan et al., 2023). The contrasting GSH responses observed among the three wheat species suggest that glutathione metabolism is regulated in a species-dependent manner rather than through a uniform cold-induced accumulation. The maintenance or slight increase of GSH under freezing conditions in *T. polonicum* may indicate a more efficient antioxidant adjustment, whereas the decline observed in *T. durum* likely reflects enhanced utilization of GSH to counteract oxidative stress. In *T. turanicum*, the relatively stable GSH levels during acclimatization and freezing suggest that redox homeostasis was maintained despite cold exposure. These findings support the significant treatment × species interaction and indicate that differences in cold response are associated with distinct antioxidant regulation strategies rather than constitutive GSH levels (Padhiar et al., 2025).

The genotype-dependent physiological and biochemical responses observed in this study are consistent with the framework proposed by Limin & Fowler (2006), which highlights that cold tolerance in wheat is shaped by genetic background and physiological adaptation processes. In this context, the better maintenance of RWC and MSI in *T. turanicum* suggests a stronger capacity to preserve cellular hydration and membrane stability under low-temperature stress. Similarly, the early proline accumulation observed in *T. turanicum* during acclimation indicates a rapid activation of osmotic adjustment mechanisms, whereas the delayed proline increase in *T. polonicum* under freezing conditions suggests a response that becomes more prominent only under severe stress. Overall, these findings indicate that low-temperature tolerance in *T. turgidum* subspecies is not governed by a single physiological or biochemical trait; rather, each genotype relies on a distinct combination of osmotic adjustment, membrane stability, pigment preservation, protein stability, and redox regulation. In line with previous reports linking higher chlorophyll content with improved cold tolerance in tetraploid wheat (Mohammadi et al., 2015), the relatively stable photosynthetic performance observed in *T. polonicum* may contribute to its ability to limit oxidative damage. In contrast, although *T. turanicum* showed marked increases in SOD, CAT, and GR activities, the concurrent high MDA levels indicate that enhanced antioxidant capacity did not fully suppress lipid peroxidation.

This supports the view that stress tolerance depends on the coordinated regulation of multiple processes rather than on antioxidant activity alone. In this regard, the genotype-specific patterns observed here are consistent with a context-dependent stress response framework (Palombieri et al., 2023). Accordingly, *T. turanicum* appears to prioritize water status and AsA-related redox buffering, whereas *T. polonicum* may rely more on maintaining photosynthetic stability and limiting oxidative damage through alternative mechanisms.

## Conclusions

In conclusion, freezing stress increased oxidative stress in all *T. turgidum* subspecies, but the response strategies were genotype-dependent. *T. turanicum* maintained a strong enzymatic antioxidant defense, whereas *T. polonicum* relied more on preserving photosynthetic stability and osmotic balance. Although *TtGR* genes were transcriptionally induced under stress, this response was not consistently reflected at the enzyme level, suggesting that mechanisms acting downstream of transcription may influence GR activity. Overall, these findings suggest that low-temperature tolerance in *T. turgidum* is determined not only by antioxidant capacity but also by the ability to sustain redox balance and cellular stability under stress conditions. However, this study is limited to controlled conditions and a restricted number of genotypes, and further validation under field conditions is required. Future studies should focus on elucidating the post-transcriptional mechanisms regulating GR activity and exploring their role in improving cold tolerance in breeding programs.

## Supplemental Information

10.7717/peerj.21678/supp-1 Supplemental Information 1 MIQE checklist.

10.7717/peerj.21678/supp-2 Supplemental Information 2 Raw data.

10.7717/peerj.21678/supp-3 Supplemental Information 3 Phylogenetic analysis of glutathione reductase (GR) proteins from *T. turgidum*, *T. aestivum*, *T*. dicoccoides, *Aegilops tauschii*, and *Arabidopsis thaliana*. The GR proteins are clearly divided into two major clades: Class I (cytosolic) and Class II (chloroplast-localized). Species are indicated by colored symbols: *T. turgidum* (green), *T. aestivum* (black), *T. dicoccoides* (blue), *A. tauschii* (gray), and *A. thaliana* (red). Chromosomal locations of wheat GR genes are shown on the right side, with subgenomes A, B, and D represented as 4A/4B/4D and 6A/6B/6D, respectively. The phylogenetic tree was constructed using the Neighbor-Joining method based on full-length protein sequences.

10.7717/peerj.21678/supp-4 Supplemental Information 4 Multiple sequence alignment of glutathione reductase (GR) proteins from *T. aestivum* (TaGR) and *T. turgidum* (TtGR). The sequences are grouped into Class I (cytosolic) and Class II (chloroplast-localized) isoforms. Conserved functional regions are indicated as follows: FAD-binding site (blue box), active site (red box), and conserved motifs (green boxes). Asterisks (*) denote fully conserved residues across all sequences. The alignment was generated using Clustal Omega.

## Additional Information and Declarations

### Data Availability

The following information was supplied regarding data availability:

The data is available in the Supplemental Files.
